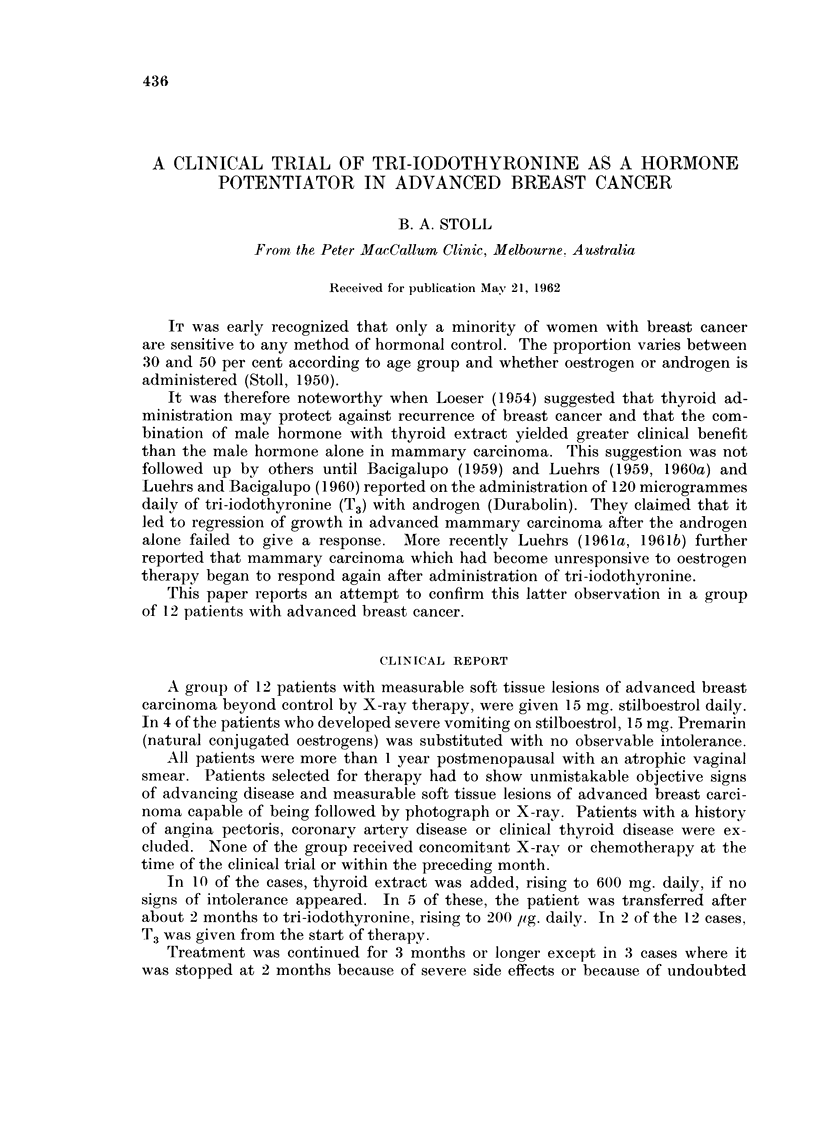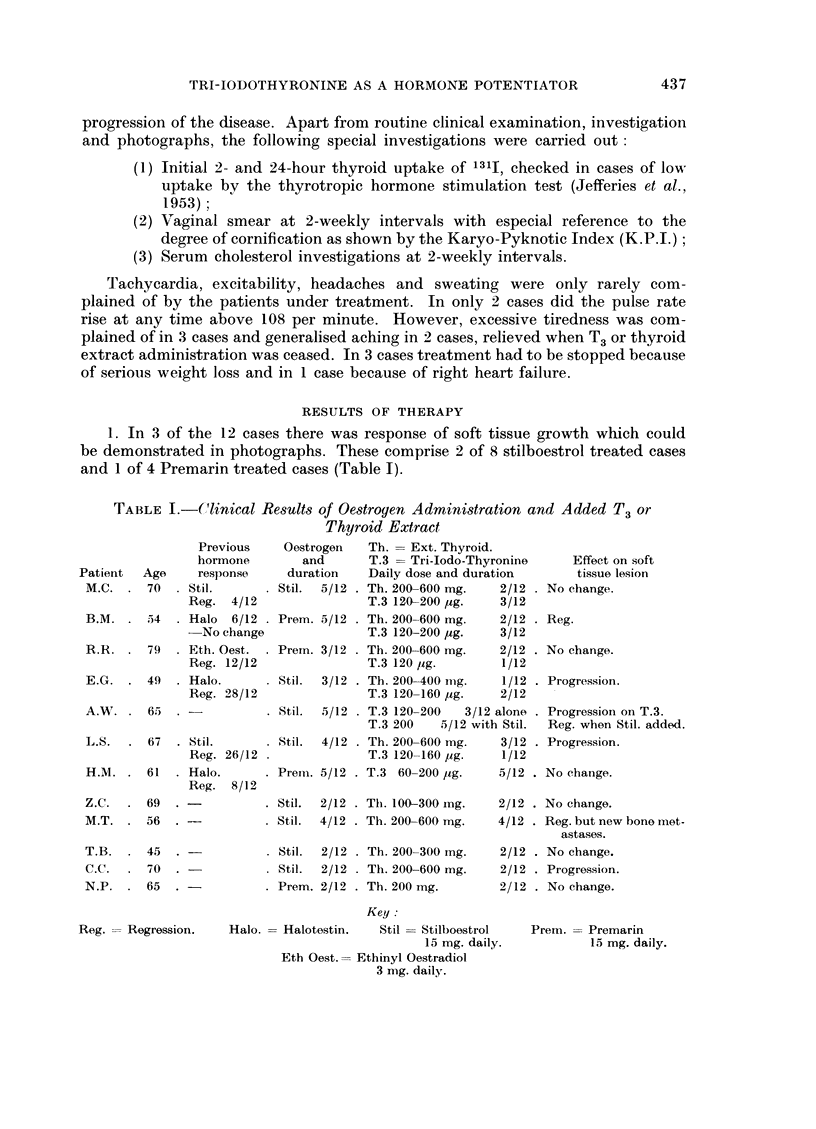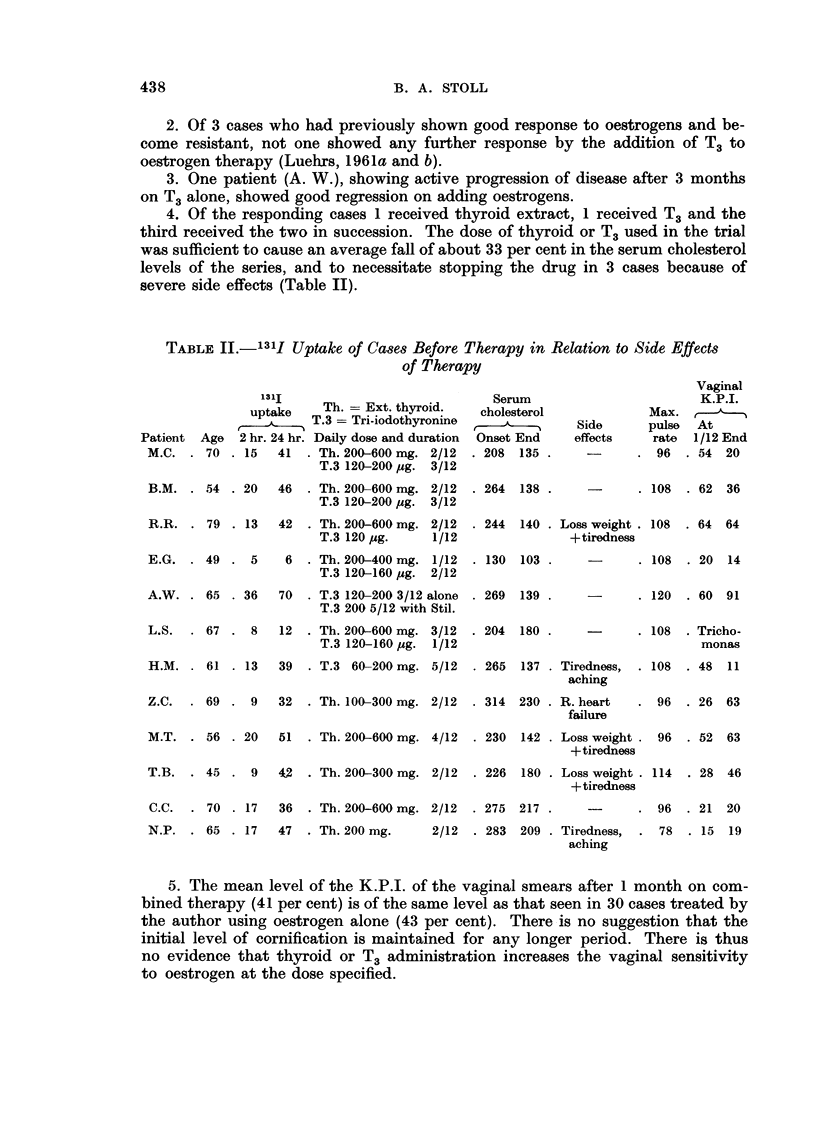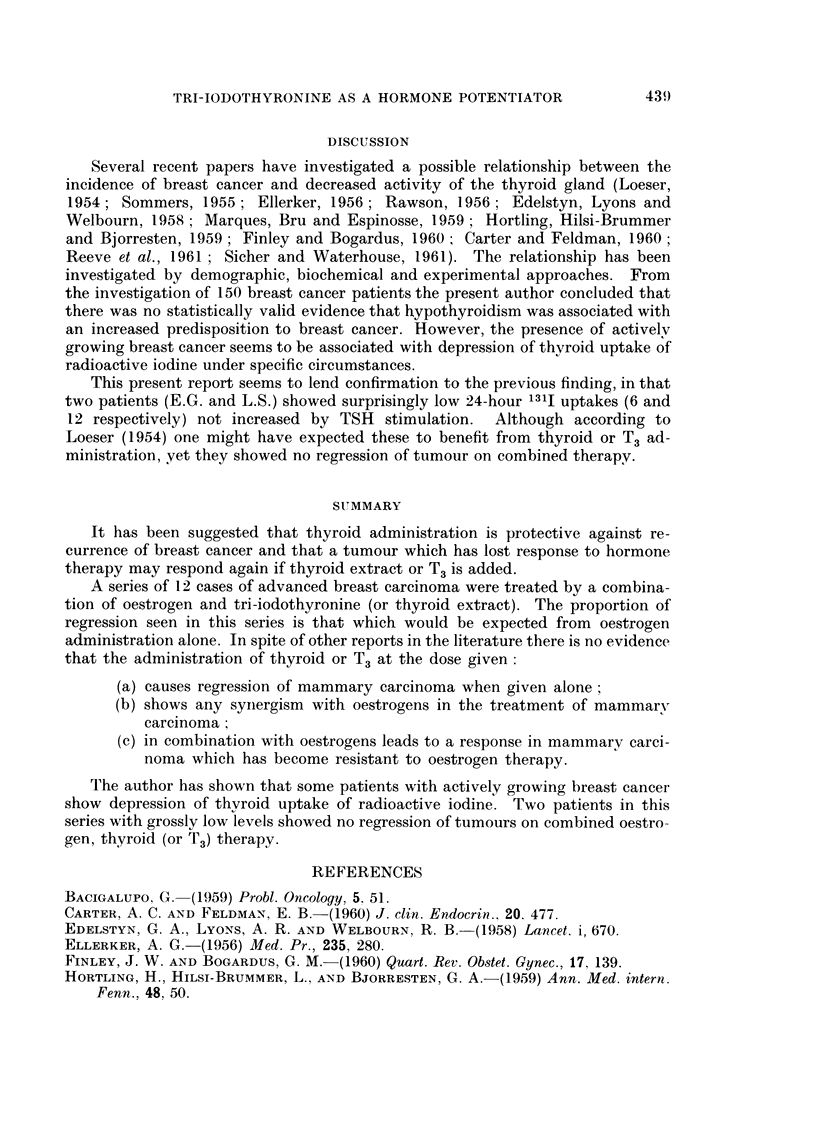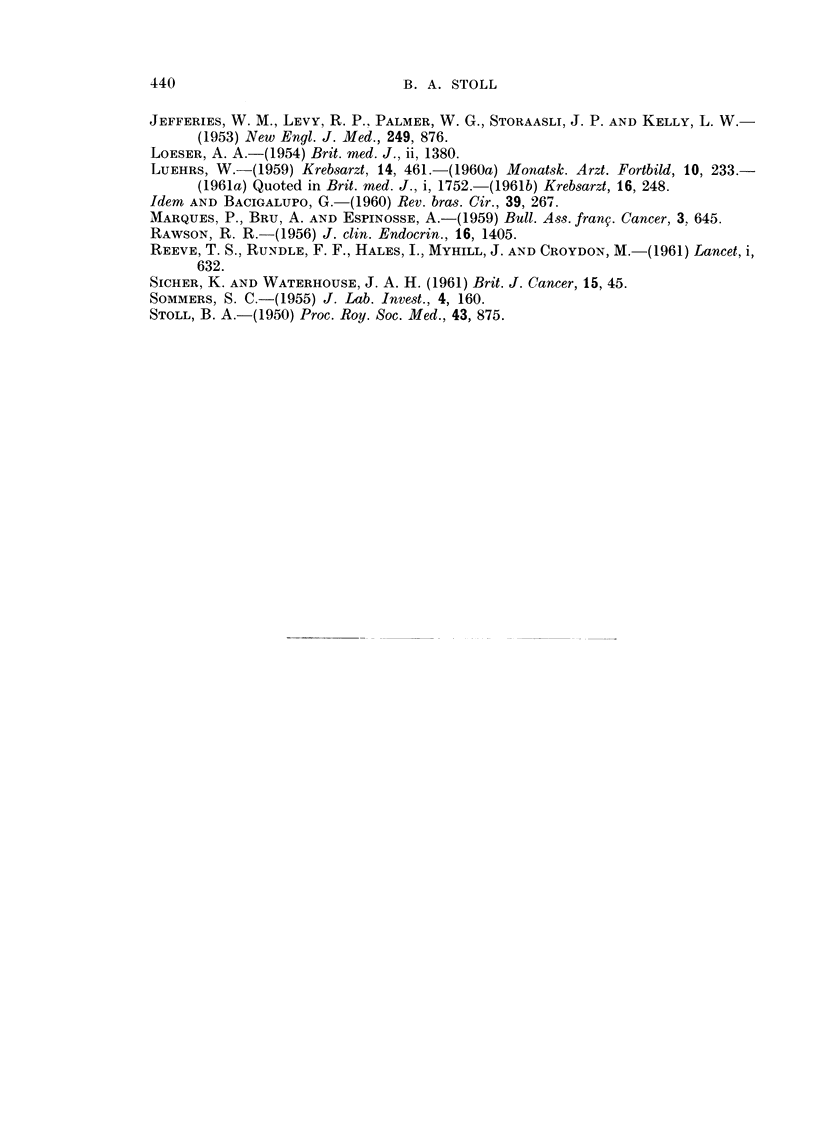# A Clinical Trial of Tri-Iodothyronine as a Hormone Potentiator in Advanced Breast Cancer

**DOI:** 10.1038/bjc.1962.48

**Published:** 1962-09

**Authors:** B. A. Stoll


### A CLINICAL TRIAL OF TRI-IODOTHYRONINE AS A HORMONE POTENTIATOR IN ADVANCED BREAST CANCER

B. A. STOLL

*From the Peter MacCallum Clinic, Melbourne, Australia*

Received for publication May 21, 1962

It was early recognized that only a minority of women with breast cancer are sensitive to any method of hormonal control. The proportion varies between 30 and 50 per cent according to age group and whether oestrogen or androgen is administered (Stoll, 1950).

It was therefore noteworthy when Loeser (1954) suggested that thyroid administration may protect against recurrence of breast cancer and that the combination of male hormone with thyroid extract yielded greater clinical benefit than the male hormone alone in mammary carcinoma. This suggestion was not followed up by others until Bacigalupo (1959) and Luehrs (1959, 1960*a*) and Luehrs and Bacigalupo (1960) reported on the administration of 120 microgrammes daily of tri-iodothyronine ($T_3$) with androgen (Durabolin). They claimed that it led to regression of growth in advanced mammary carcinoma after the androgen alone failed to give a response. More recently Luehrs (1961*a*, 1961*b*) further reported that mammary carcinoma which had become unresponsive to oestrogen therapy began to respond again after administration of tri-iodothyronine.

This paper reports an attempt to confirm this latter observation in a group of 12 patients with advanced breast cancer.

### CLINICAL REPORT

A group of 12 patients with measurable soft tissue lesions of advanced breast carcinoma beyond control by X-ray therapy, were given 15 mg. stilboestrol daily. In 4 of the patients who developed severe vomiting on stilboestrol, 15 mg. Premarin (natural conjugated oestrogens) was substituted with no observable intolerance.

All patients were more than 1 year postmenopausal with an atrophic vaginal smear. Patients selected for therapy had to show unmistakable objective signs of advancing disease and measurable soft tissue lesions of advanced breast carcinoma capable of being followed by photograph or X-ray. Patients with a history of angina pectoris, coronary artery disease or clinical thyroid disease were excluded. None of the group received concomitant X-ray or chemotherapy at the time of the clinical trial or within the preceding month.

In 10 of the cases, thyroid extract was added, rising to 600 mg. daily, if no signs of intolerance appeared. In 5 of these, the patient was transferred after about 2 months to tri-iodothyronine, rising to 200 $\mu$g. daily. In 2 of the 12 cases, $T_3$ was given from the start of therapy.

Treatment was continued for 3 months or longer except in 3 cases where it was stopped at 2 months because of severe side effects or because of undoubted progression of the disease. Apart from routine clinical examination, investigation and photographs, the following special investigations were carried out :

(1) Initial 2- and 24-hour thyroid uptake of $^{131}I$, checked in cases of low uptake by the thyrotropic hormone stimulation test (Jefferies *et al.*, 1953) ;
(2) Vaginal smear at 2-weekly intervals with especial reference to the degree of cornification as shown by the Karyo-Pyknotic Index (K.P.I.) ;
(3) Serum cholesterol investigations at 2-weekly intervals.

Tachycardia, excitability, headaches and sweating were only rarely complained of by the patients under treatment. In only 2 cases did the pulse rate rise at any time above 108 per minute. However, excessive tiredness was complained of in 3 cases and generalised aching in 2 cases, relieved when $T_3$ or thyroid extract administration was ceased. In 3 cases treatment had to be stopped because of serious weight loss and in 1 case because of right heart failure.

### RESULTS OF THERAPY

1. In 3 of the 12 cases there was response of soft tissue growth which could be demonstrated in photographs. These comprise 2 of 8 stilboestrol treated cases and 1 of 4 Premarin treated cases (Table I).

TABLE I.—*Clinical Results of Oestrogen Administration and Added $T_3$ or Thyroid Extract*

| Patient | Age | Previous hormone response | Oestrogen and duration | Th. = Ext. Thyroid. T.3 = Tri-Iodo-Thyronine Daily dose and duration | Effect on soft tissue lesion |
|---|---|---|---|---|---|
| M.C. | 70 | Stil. Reg. 4/12 | Stil. 5/12 | Th. 200–600 mg. 2/12<br>T.3 120–200 μg. 3/12 | No change. |
| B.M. | 54 | Halo 6/12 —No change | Prem. 5/12 | Th. 200–600 mg. 2/12<br>T.3 120–200 μg. 3/12 | Reg. |
| R.R. | 79 | Eth. Oest. Reg. 12/12 | Prem. 3/12 | Th. 200–600 mg. 2/12<br>T.3 120 μg. 1/12 | No change. |
| E.G. | 49 | Halo. Reg. 28/12 | Stil. 3/12 | Th. 200–400 mg. 1/12<br>T.3 120–160 μg. 2/12 | Progression. |
| A.W. | 65 | — | Stil. 5/12 | T.3 120–200 3/12 alone<br>T.3 200 5/12 with Stil. | Progression on T.3. Reg. when Stil. added. |
| L.S. | 67 | Stil. Reg. 26/12 | Stil. 4/12 | Th. 200–600 mg. 3/12<br>T.3 120–160 μg. 1/12 | Progression. |
| H.M. | 61 | Halo. Reg. 8/12 | Prem. 5/12 | T.3 60–200 μg. 5/12 | No change. |
| Z.C. | 69 | — | Stil. 2/12 | Th. 100–300 mg. 2/12 | No change. |
| M.T. | 56 | — | Stil. 4/12 | Th. 200–600 mg. 4/12 | Reg. but new bone metastases. |
| T.B. | 45 | — | Stil. 2/12 | Th. 200–300 mg. 2/12 | No change. |
| C.C. | 70 | — | Stil. 2/12 | Th. 200–600 mg. 2/12 | Progression. |
| N.P. | 65 | — | Prem. 2/12 | Th. 200 mg. 2/12 | No change. |

*Key :*

Reg. = Regression. Halo. = Halotestin. Stil = Stilboestrol 15 mg. daily. Prem. = Premarin 15 mg. daily.

Eth Oest. = Ethinyl Oestradiol 3 mg. daily.

2. Of 3 cases who had previously shown good response to oestrogens and become resistant, not one showed any further response by the addition of $T_3$ to oestrogen therapy (Luehrs, 1961*a* and *b*).

3. One patient (A. W.), showing active progression of disease after 3 months on $T_3$ alone, showed good regression on adding oestrogens.

4. Of the responding cases 1 received thyroid extract, 1 received $T_3$ and the third received the two in succession. The dose of thyroid or $T_3$ used in the trial was sufficient to cause an average fall of about 33 per cent in the serum cholesterol levels of the series, and to necessitate stopping the drug in 3 cases because of severe side effects (Table II).

TABLE II.—$^{131}I$ *Uptake of Cases Before Therapy in Relation to Side Effects of Therapy*

| Patient | Age | $^{131}$I uptake 2 hr. | 24 hr. | Th. = Ext. thyroid. T.3 = Tri-iodothyronine Daily dose and duration | Serum cholesterol Onset | End | Side effects | Max. pulse rate | Vaginal K.P.I. At 1/12 | End |
|---|---|---|---|---|---|---|---|---|---|---|
| M.C. | 70 | 15 | 41 | Th. 200–600 mg. 2/12<br>T.3 120–200 μg. 3/12 | 208 | 135 | — | 96 | 54 | 20 |
| B.M. | 54 | 20 | 46 | Th. 200–600 mg. 2/12<br>T.3 120–200 μg. 3/12 | 264 | 138 | — | 108 | 62 | 36 |
| R.R. | 79 | 13 | 42 | Th. 200–600 mg. 2/12<br>T.3 120 μg. 1/12 | 244 | 140 | Loss weight +tiredness | 108 | 64 | 64 |
| E.G. | 49 | 5 | 6 | Th. 200–400 mg. 1/12<br>T.3 120–160 μg. 2/12 | 130 | 103 | — | 108 | 20 | 14 |
| A.W. | 65 | 36 | 70 | T.3 120–200 3/12 alone<br>T.3 200 5/12 with Stil. | 269 | 139 | — | 120 | 60 | 91 |
| L.S. | 67 | 8 | 12 | Th. 200–600 mg. 3/12<br>T.3 120–160 μg. 1/12 | 204 | 180 | — | 108 | Trichomonas | |
| H.M. | 61 | 13 | 39 | T.3 60–200 mg. 5/12 | 265 | 137 | Tiredness, aching | 108 | 48 | 11 |
| Z.C. | 69 | 9 | 32 | Th. 100–300 mg. 2/12 | 314 | 230 | R. heart failure | 96 | 26 | 63 |
| M.T. | 56 | 20 | 51 | Th. 200–600 mg. 4/12 | 230 | 142 | Loss weight +tiredness | 96 | 52 | 63 |
| T.B. | 45 | 9 | 42 | Th. 200–300 mg. 2/12 | 226 | 180 | Loss weight +tiredness | 114 | 28 | 46 |
| C.C. | 70 | 17 | 36 | Th. 200–600 mg. 2/12 | 275 | 217 | — | 96 | 21 | 20 |
| N.P. | 65 | 17 | 47 | Th. 200 mg. 2/12 | 283 | 209 | Tiredness, aching | 78 | 15 | 19 |

5. The mean level of the K.P.I. of the vaginal smears after 1 month on combined therapy (41 per cent) is of the same level as that seen in 30 cases treated by the author using oestrogen alone (43 per cent). There is no suggestion that the initial level of cornification is maintained for any longer period. There is thus no evidence that thyroid or $T_3$ administration increases the vaginal sensitivity to oestrogen at the dose specified.

### DISCUSSION

Several recent papers have investigated a possible relationship between the incidence of breast cancer and decreased activity of the thyroid gland (Loeser, 1954; Sommers, 1955; Ellerker, 1956; Rawson, 1956; Edelstyn, Lyons and Welbourn, 1958; Marques, Bru and Espinosse, 1959; Hortling, Hilsi-Brummer and Bjorresten, 1959; Finley and Bogardus, 1960; Carter and Feldman, 1960; Reeve *et al.*, 1961; Sicher and Waterhouse, 1961). The relationship has been investigated by demographic, biochemical and experimental approaches. From the investigation of 150 breast cancer patients the present author concluded that there was no statistically valid evidence that hypothyroidism was associated with an increased predisposition to breast cancer. However, the presence of actively growing breast cancer seems to be associated with depression of thyroid uptake of radioactive iodine under specific circumstances.

This present report seems to lend confirmation to the previous finding, in that two patients (E.G. and L.S.) showed surprisingly low 24-hour $^{131}I$ uptakes (6 and 12 respectively) not increased by TSH stimulation. Although according to Loeser (1954) one might have expected these to benefit from thyroid or $T_3$ administration, yet they showed no regression of tumour on combined therapy.

### SUMMARY

It has been suggested that thyroid administration is protective against recurrence of breast cancer and that a tumour which has lost response to hormone therapy may respond again if thyroid extract or $T_3$ is added.

A series of 12 cases of advanced breast carcinoma were treated by a combination of oestrogen and tri-iodothyronine (or thyroid extract). The proportion of regression seen in this series is that which would be expected from oestrogen administration alone. In spite of other reports in the literature there is no evidence that the administration of thyroid or $T_3$ at the dose given:

(a) causes regression of mammary carcinoma when given alone;
(b) shows any synergism with oestrogens in the treatment of mammary carcinoma;
(c) in combination with oestrogens leads to a response in mammary carcinoma which has become resistant to oestrogen therapy.

The author has shown that some patients with actively growing breast cancer show depression of thyroid uptake of radioactive iodine. Two patients in this series with grossly low levels showed no regression of tumours on combined oestrogen, thyroid (or $T_3$) therapy.

### REFERENCES

BACIGALUPO, G.—(1959) *Probl. Oncology*, **5**, 51.
CARTER, A. C. AND FELDMAN, E. B.—(1960) *J. clin. Endocrin.*, **20**, 477.
EDELSTYN, G. A., LYONS, A. R. AND WELBOURN, R. B.—(1958) *Lancet*, i, 670.
ELLERKER, A. G.—(1956) *Med. Pr.*, **235**, 280.
FINLEY, J. W. AND BOGARDUS, G. M.—(1960) *Quart. Rev. Obstet. Gynec.*, **17**, 139.
HORTLING, H., HILSI-BRUMMER, L., AND BJORRESTEN, G. A.—(1959) *Ann. Med. intern. Fenn.*, **48**, 50.

JEFFERIES, W. M., LEVY, R. P., PALMER, W. G., STORAASLI, J. P. AND KELLY, L. W.—(1953) *New Engl. J. Med.*, **249**, 876.
LOESER, A. A.—(1954) *Brit. med. J.*, ii, 1380.
LUEHRS, W.—(1959) *Krebsarzt*, **14**, 461.—(1960*a*) *Monatsk. Arzt. Fortbild*, **10**, 233.—(1961*a*) Quoted in *Brit. med. J.*, i, 1752.—(1961*b*) *Krebsarzt*, **16**, 248.
*Idem* AND BACIGALUPO, G.—(1960) *Rev. bras. Cir.*, **39**, 267.
MARQUES, P., BRU, A. AND ESPINOSSE, A.—(1959) *Bull. Ass. franç. Cancer*, **3**, 645.
RAWSON, R. R.—(1956) *J. clin. Endocrin.*, **16**, 1405.
REEVE, T. S., RUNDLE, F. F., HALES, I., MYHILL, J. AND CROYDON, M.—(1961) *Lancet*, i, 632.
SICHER, K. AND WATERHOUSE, J. A. H. (1961) *Brit. J. Cancer*, **15**, 45.
SOMMERS, S. C.—(1955) *J. Lab. Invest.*, **4**, 160.
STOLL, B. A.—(1950) *Proc. Roy. Soc. Med.*, **43**, 875.